# Considerations for Nurses in Recommending mHealth Apps for People Living With Chronic Conditions: A Qualitative Study

**DOI:** 10.1002/nop2.70598

**Published:** 2026-07-01

**Authors:** Wa'ed Shiyab, Kaye Rolls, Caleb Ferguson, Elizabeth Halcomb

**Affiliations:** ^1^ School of Nursing, Faculty of Science, Medicine & Health University of Wollongong Wollongong New South Wales Australia; ^2^ Centre for Chronic & Complex Care Research, Blacktown Hospital, Western Sydney Local Health District & School of Nursing, Faculty of Science, Medicine & Health University of Wollongong Wollongong New South Wales Australia

**Keywords:** chronic conditions, mHealth app, nurses, qualitative research

## Abstract

**Background:**

Mobile Health (mHealth) applications are innovative tools that have contributed to healthcare digital transformation. While healthcare professionals positively perceive the role of mHealth in improving patients' health outcomes, embedding mHealth app recommendations into routine clinical practice is still in its infancy. There is a limited understanding of the factors nurses consider when recommending mHealth apps.

**Aim:**

This paper explores the factors affecting the recommendation of mHealth apps by nurses caring for people living with or at risk of chronic conditions.

**Design:**

Qualitative descriptive study within a concurrent mixed methods project.

**Methods:**

Semi‐structured videoconference interviews were conducted with 13 nurses. Interviews were audio‐recorded, transcribed verbatim, and analysed using thematic analysis. The COREQ checklist guided reporting.

**Results:**

Two main themes were identified: (1) clinical considerations and (2) technical considerations. Clinical considerations revealed how credibility and trustworthiness, accessibility, and personalisation impacted recommendations around mHealth apps. Conversely, technical considerations identified factors related to the design, functionalities, and implementation of mHealth apps, namely, data security and privacy, usability, app overload and interoperability.

**Conclusion:**

Understanding the factors that influence nurses' recommendations of mHealth apps is essential to inform future integration into chronic disease management. Considerations include ensuring patients have reliable access, customising apps to meet users' preferences and needs, and linking them with electronic medical records. mHealth apps should have a user‐friendly interface and provide up‐to‐date, evidence‐based content.

**Relevance to Clinical Practice:**

Factors nurses consider when recommending mHealth apps can affect how they integrate them into clinical practice. Understanding these factors can inform strategies to enhance nurses' integration of mHealth apps in chronic condition management.

**Patient or Public Contribution:**

No patient or public contribution.

## 1 Introduction

Globally, healthcare systems are experiencing growing demands for services due to the prevalence of chronic conditions, combined with an ageing population, pandemics and increasing socioeconomic disparities (World Health Organization 2020). Many chronic conditions can be prevented or their progression slowed by changing health behaviours to reduce lifestyle risk (Rahelic et al. 2024). Increasing physical activity, eating a healthy diet, ceasing smoking, and reducing alcohol intake are key lifestyle changes that can reduce both risk and progression across a variety of chronic conditions (Rahelic et al. 2024). Achieving such change requires support from health professionals for lifestyle modification and behavioural change (Matthews et al. 2024). Given the increasing demands on health services, developing new and innovative approaches to manage health service delivery is vital.

Digital technologies have transformed the delivery of and access to healthcare (Stoumpos et al. 2023). Mobile Health (mHealth) applications (apps) are software on mobile devices that support health and well‐being (Maaß et al. 2022). In particular, apps can facilitate self‐management by providing a platform for tracking symptoms (e.g., menstruation, shortness of breath), recording biometric values (e.g., weight, blood sugar) or behaviour patterns (e.g., sleep, physical activity), and providing health education materials (Wells and Spry 2022). Both patients and clinicians can utilise apps, and they also offer a way to remotely communicate between patients and clinicians, or provide patient data to clinicians to inform care (Wells and Spry 2022).

mHealth apps have become increasingly popular, especially following the COVID‐19 pandemic (Yang et al. 2023). In recent years, there has been a significant increase in the number of downloads of apps related to exercise, mental health, and the management of chronic conditions, such as diabetes and cardiovascular disease (IQVIA Institute for Human Data Science 2021). mHealth apps have the potential to assist in tracking and influencing behaviours to enhance preventative health practices, promote lifestyle change and help manage chronic conditions. For example, using mHealth apps can help people achieve better glycaemic control in diabetes management, reduce the incidence of cardiac events, improve medication adherence, and enhance cardiac function (Tu et al. 2021; Zhu et al. 2024). However, there is a need to involve healthcare professionals in using mHealth apps to optimise their impact (Shiyab et al. 2023). While healthcare professionals have a positive perception of the role of mHealth in improving patients' health outcomes (Shiyab et al. 2024), embedding the recommendation of mHealth apps into patients' usual clinical practice is still in its infancy (Byambasuren et al. 2019). Additionally, the uptake of mHealth apps in practice by nurses has been reported as being low (de Jong et al. 2020; Shiyab et al. 2024).

The Unified Theory of Acceptance and Use of Technology (UTAUT) provides a theoretical framework to understand app use. The UTAUT integrates theories and models from sociology, psychology, and communication to form a unified model (Taherdoost 2018; Venkatesh et al. 2003). The UTAUT and its extensions have been widely used to guide studies that explore the acceptance of technology in healthcare (AlQudah et al. 2021). The four constructs of the UTAUT can predict nurses' behaviour towards mHealth apps. Performance Expectancy refers to the perception of increased productivity through using mHealth apps, while Effort Expectancy is the perception of how complex it is to use mHealth apps (Venkatesh et al. 2003). Subsequently, Social Influence involves the perception of others' influence on the use of mHealth apps and Facilitating Conditions pertain to the perception of the amount of technical support available to address issues that arise from using mHealth apps (Venkatesh et al. 2003).

Previous studies have reported various ethical, practical, and socio‐technical factors affecting the recommendation of mHealth apps among healthcare professionals (Byambasuren et al. 2020; Leigh et al. 2020; Sarradon‐Eck et al. 2021). However, most studies undertaken to date have focused on physicians (Byambasuren et al. 2020; Sarradon‐Eck et al. 2021), on specific specialties (Kayyali et al. 2017) or had a multidisciplinary sample (Leigh et al. 2020). Leigh et al. (2020) found that healthcare providers have different drivers towards recommending mHealth apps, suggesting that using a single approach to increase digital uptake among healthcare professionals may be ineffective. Therefore, research is needed with specific groups of health professionals, such as nurses, to understand their unique concerns and drivers for recommending mHealth apps.

The limited literature available on mHealth app use among nurses makes it challenging to understand the issues surrounding nurses and the recommendations of mHealth apps. Given that nursing is the largest healthcare professional group (World Health Organization 2025) and nurses are trusted by patients to provide health advice and self‐management support (Ashley et al. 2020), it seems appropriate for nurses to integrate the technological advances offered by mHealth apps in the self‐management of chronic disease (Ferguson and Jackson 2017). A key step in encouraging nurses to integrate mHealth apps into their daily care routine and recommend them to patients is to understand the factors nurses consider when recommending mHealth apps.

## 2 Methods

### 2.1 Aim

This paper explores the factors affecting the recommendation of mHealth apps by nurses caring for people living with or at risk of chronic conditions. Given the limited literature about mHealth app use among nurses and the similarities of lifestyle/behaviour change across conditions, a broad approach was chosen to gain a view of the issue across chronic conditions.

### 2.2 Design

Findings are drawn from the qualitative study of a larger concurrent mixed‐method project. The quantitative survey explored nurses' use of mHealth apps to support adults with or at risk of chronic conditions (Shiyab et al. 2024). In the qualitative study, semi‐structured interviews were conducted with a subgroup of survey respondents using a qualitative descriptive approach to further explore the phenomenon (Doyle et al. 2020). This study was reported following the guidelines outlined in the Consolidated Criteria for Reporting Qualitative Research (COREQ) (Tong et al. 2007).

### 2.3 Participants and Recruitment

Australian nurses who self‐identified as providing direct care for patients with or at risk of chronic conditions were eligible to respond to the initial survey. This included diploma‐prepared enrolled nurses, baccalaureate‐prepared registered nurses, or Master's‐prepared nurse practitioners. From the survey, thirty‐five respondents indicated an interest in participating in the interviews. Information about the interview and a consent form were emailed to these potential participants, and they were followed up with up to three reminder emails. Twenty‐two people either declined or did not respond to this invitation, leaving 13 people willing to participate in the interviews. Verbal consent was sought via Qualtrics. Given the modest number, all 13 were interviewed.

### 2.4 Data Collection

Data were collected using semi‐structured individual interviews. An interview schedule was developed from initial survey data (Shiyab et al. 2024), a review of the literature and opinion from nurse researchers with expertise in use of technology/mHealth. The schedule included open‐ended questions, such as, ‘What influences your use of apps for people living with chronic conditions?’, ‘What are your thoughts about nurses recommending apps for people with chronic conditions?’ Probes and prompts were used to elicit deeper information, for example, ‘How did you decide to use the app?, What were the challenges?’

The first author (WS), a female registered nurse holding a master's degree in nursing and trained in qualitative interviewing methods, conducted the interviews from December 2022 to February 2023. Zoom Video conference interviews were used due to the geographical spread of participants and COVID‐19 restrictions (Saarijärvi and Bratt 2021). The interviews were audio recorded and transcribed verbatim by a professional transcription company, and field notes were kept. Given the additional burden it would impose on participants and the limited personal value, transcripts were not returned to participants for member‐checking.

### 2.5 Data Analysis

Transcripts were managed using NVivo software, and data were analysed using the thematic analysis framework described by Braun and Clarke (2022). Data analysis was an iterative process discussed between the four researchers to gain consensus. In addition to the PhD candidate, the other researchers were experienced nurse researchers with expertise in chronic condition management and the use of technology in health. First, the PhD candidate immersed herself in the data by reading and rereading the transcripts and listening to the audio recordings. Initial notes were taken at this stage to reflect on patterns in the data. By labelling meaningful units of the transcripts, initial codes were systematically generated. The next phase involved grouping similar codes into themes. The initial themes were reviewed by checking whether the coded data extracts formed a coherent pattern and whether these themes represented the data and addressed the research questions. Initial themes were revised either by collapsing some themes together or by deletion. A consensus was reached between all researchers on the final themes. Finally, the report was written to show the reader how the themes represent the data.

### 2.6 Ethical Considerations

The study was approved by the Human Research Ethics Committee of the University of Wollongong (Approval No. 2022/202) prior to commencement. Participants were emailed the information sheet, which included the study purpose, researchers, potential risks, and participants' rights, and they were advised that they could cease the interview at any time. To protect participants' confidentiality, pseudonyms are used in reporting.

### 2.7 Rigour

The principles described by Lincoln and Guba (1985) were employed to enhance the trustworthiness and rigour of the study. Credibility was achieved by checking the transcripts against the recordings, ensuring the accuracy of participants' responses. The research team also validated the findings to minimise subjectivity. Confirmability was established by audio recording the interviews, taking field notes and incorporating them to capture the full context of the data. Providing rich descriptions of the participants and research context helped to establish transferability and helped to assess the applicability of the findings in different settings. To ensure dependability, the same interview guide was used for all participants, and an audit trail was maintained by documenting the data collection and analysis steps.

## 3 Results

Most of the 13 participants (*n* = 12; 92.3%) were female. Participants' mean age was 47.5 years (28–64 years). More than half (*n* = 7; 53.8%) of the participants were registered nurses, four participants (30.8%) were clinical nurse consultants, and the other two (15.4%) were a nurse practitioner and nurse educator. Five participants (38.4%) worked in general practice, three (23.1%) in outpatient services, three (23.1%) in the community, one (7.7%) in a correctional centre, and one (7.7%) in a hospital. The majority of the participants (*n* = 9, 69.2%) were from metropolitan areas, three (23.1%) from rural areas, and one (7.7%) from a remote area. The interview duration ranged from 19 to 53 min (mean 27 min).

Two themes were identified regarding factors affecting mHealth app recommendation: (1) clinical considerations and (2) technical considerations. The themes and their subthemes are shown in Table 1.

**TABLE 1:** Thematic structure.

Theme	Subthemes
Clinical considerations	mHealth app credibility and trustworthiness Accessibility Personalisation
2 Technical considerations	Data security and privacy Usability mHealth app overload mHealth app interoperability

### 3.1 Clinical Considerations

Clinical considerations encompassed nurses' perceptions of the factors that influence mHealth app recommendations in their clinical practice and could be divided into three sub‐themes: (a) credibility and trustworthiness, (b) accessibility, and (c) personalisation.

- Credibility and Trustworthiness

Participants identified the perceived credibility of mHealth apps as an important consideration when recommending them. Some participants described being more likely to recommend mHealth apps with a clear clinical foundation or if trusted healthcare providers developed their information.

I think if I was to recommend the apps to patients with chronic conditions, I'd want to be sure that app sat with some sort of clinical acumen. So, who invented it, and why did they invent it? That's why I like that cancer app out of *[teaching hospital]* because it was invented by doctors working with patients with cancer. (Felix)

And I'll say, well, they've done it in conjunction with the university to create this app or different things like that so that there's some sort of study behind it. (Nilly)

Other participants agreed that mHealth apps would be more trusted if they were endorsed by a trusted group or peak body (such as the Heart Foundation or the Australian Diabetes Educators Association).

I think it's; it would also be good if the hospital itself will have some apps that they would want us to recommend. And if they're screened by the hospital, that would be a lot better. (Irene)

But if they (mHealth apps) come from the foundations themselves, then I'd be more likely to recommend them and more likely to endorse them. (Kate)

Participants also highlighted the importance of apps maintaining their contemporary status, whereby content was updated regularly as evidence matures.

As long as they're updated regularly, and that's what I mean by if you're going to have, use your apps for best practice, evidence‐based practice, then somebody's got to maintain that evidence on the app. (Layla)

b Accessibility

Participants described the accessibility of mHealth apps as an important consideration. Accessibility factors included the cost of the app and any devices for the patient, as well as access to the internet and a power source.

Several participants described the cost of mHealth apps as a barrier to their recommendation. Emelia explained that *‘nobody likes spending any money’*. Felix stated that ‘*app technology remains consistently expensive*’ and Kate said that ‘*cost is probably going to be a barrier*’ to the accessibility of mHealth apps. Further, Maddy explained that ‘*the cost of technology isn't reimbursed by Medicare, which then creates an inequity for people who can't afford private health cover*’. Layla suggested that funding patients to access technology ‘*would be a cost‐effective way of doing healthcare’*. She added, ‘*If we want people to use technology, we might have to provide them with the devices they can use to access that technology… and help them engage with their chronic disease*’.

Beyond the cost of the mHealth app, *‘the cost [*of a device*] is a significant challenge’* (Daisy), or ‘*not everyone has a phone. We assume they do, but they don't’* (Helen). Layla discussed how this limited access to technology, particularly for those who were socio‐economically disadvantaged: *‘The challenge is that app relies on a phone, basically, a good working android or iPhone. So, for people that have got financial issues and don't have access to technology’*. Even for those with a device, *‘if their equipment hasn't updated, some of them might have a phone that can't handle a particular app’* (Nilly). Maddy agreed, highlighting the challenge if ‘*the device is not compatible… that's a huge issue with Android devices because there are so many of them’*.

Kate described that if patients already owned suitable devices, it would encourage her to recommend mHealth apps. *‘If they have a smartwatch, I recommend they look at their heart rate and monitor it’*. Abi agreed, *‘Perhaps if they didn't have a visible watch and I couldn't see that they had a smartphone, it would be harder for me to suggest because I would be worried that they wouldn't have the capacity to actually access this… But I could see that they were already equipped at the time’*.

c Personalisation

Participants described how mHealth apps are typically designed for a specific population, such as those of a young age and those who are English‐speaking, which may limit their appropriateness for a broader population. Felix stated;

When I think about mobile app technology and chronic conditions in older patients, is that end usability? So, the app might have been designed by a 20‐year‐old and built by a 20‐year‐old, but if your target audience is over 65, how much user experience and usability testing was there? So, for example, does it support languages other than English? Can you change the font size to larger?

Caroline explained, ‘I think it all (mHealth apps) assumes we speak good English and can read English, which we don't… Some of the terms I think are too medical or they're too technical’.

Healthcare providers' perceptions of older adults were also considered important. Abi explained, ‘*To be perfectly honest, I don't have very much experience – if at all – recommending health apps to patients because I think, number one, a lot of the patients…. are of the older population.’* In contrast, Caroline described that comments about older people not wanting to use mHealth apps may result from stereotyping and nurses' bias rather than their experience. *‘I think the assumption that people don't want to do it. I have some old patients who love their technology. But if you looked at them – so that's like a bias, ageism, the nurses' own stereotyping’*.

Many participants highlighted the importance of assessing individual patients' needs, interests, and abilities. Grace described a need for *‘being cautious of overwhelming people with having to enter data and it becomes quite stressful because they think if they miss entering in stuff then it's going to be of no use, and then or they get alerts and the alerts become too overwhelming and it's interfering with their daily life or their sleep at night’*. Similarly, Belinda suggested how *‘we need to ask the person, ‘Are you interested in this? Is this something that you would find helpful?’ because it's not just up to us as nurses to push that onto them’*.

### 3.2 Technical Considerations

The theme of technical considerations includes factors related to the design, functionalities, and implementation of mHealth apps and can be divided into four subthemes: (a) data security and privacy, (b) usability, (c) mHealth app overload, and (d) mHealth app interoperability

- Data Security and Privacy

Participants described concerns about data security and privacy within mHealth apps. *‘The biggest barrier for me, number one, is… where is my data going? Like is this safe? Is this private?’* (Abi). Similarly, Felix explained that *‘one of the other worries is that we're not sure where the source of information is and where our data is stored’*. While *‘the technology might exist…. my workplace legislation hasn't caught up. The privacy act, the privacy act was written in 1988, right? In 1988, mobile phone technology was nothing like it is today’ (Felix)*. These concerns made participants reluctant to recommend mHealth apps.

b Usability

Participants considered app usability an important factor to consider before recommending them to patients. Layla described it as if ‘*it's intuitive, it's easy to use, it's easy to navigate, then patients are going to engage with it*.’ Conversely, participants highlighted how some mHealth app features like unnecessary questions, frequent alarms, and multiple authentication, made them complicated and not user‐friendly.

Recently, I tried one that was about meditation and mindfulness, and when I logged in, the first thing it asked me was lots of demographic questions, what my gender was, and what I identify as and what my – things that I didn't feel were important to the role of the app. And that will turn me off an app straightaway. (Felix)

So, you have to be quite cautious perhaps with alarms and how many alarms they're getting as well. So, they're probably the main issues. (Grace)

The hardest part for me was this authentication within the app; I think that's a real issue. (Felix)

Such perceptions about ease of use acted as a barrier for participants to recommend apps to their patients.

c mHealth Apps Overload

Many participants described the overabundance of mHealth apps as a challenge because ‘*it's hard to choose which ones (mHealth apps) will work for you… There's so many*’ (Abi). Grace agreed, *‘and that's why I've gone now to just looking at apps that people recommend or clients who come in recommend; they're* (mHealth apps) *the ones I look at now instead of searching or doing anything. Because there are so many out there’*. Similarly, Maddy commented, ‘There's *research that's been done on something like 8000 apps, and only a very small number, something like 20%, had actually been tested and validated*’.

Felix raised concerns about the number of apps needed if people have multiple chronic conditions. He stated, *‘I think one of the risks there is if I had COPD, diabetes, some high blood pressure, some heart disease, I'd had a stroke, do I now need five or six apps for each one of those chronic conditions?’* Kate agreed, *‘So, I generally have multiple apps for different things, but there would be a significant overlap in function of each of those apps, but none of them do everything’*. Grace suggested that ‘*it would be nice to have everything incorporated in one app, which is something that would be fantastic’*.

d mHealth App Interoperability

Several participants questioned the benefits of mHealth app data if it were not shared or connected with the healthcare provider, the patient's electronic health record or existing digital health infrastructure. Felix discussed that the data is ‘*virtually useless if you're the clinician and you don't see it or own it; you can't do anything with it’*. He added, *‘I have never seen extraction of that data that's been useful’*. Emelia thought, ‘*Link a service to your app would be good like link My Health Record or link my chemistry, my medication list on there’*.

In contrast, other participants described how some mHealth apps could be better linked to their clinical systems, making them a helpful tool to inform timely intervention. Layla explained, *‘I can stop something bad happening. I can stop a hypo. So, I can assist the patient to make a decision about what they need to do before they have an acute episode of something’*. Maddy added further benefits like facilitating healthcare delivery despite geographical distance and increasing the efficacy of appointments.

Have done some work in remote areas, and that was a fly‐in, fly‐out situation where I would visit once a month. And sometimes people need support. So, the ability to use an app to communicate, to collect the information about a CGM firstly, and I can then look at that data from my computer wherever I am. So, downloading all of that data is automated; it means that I can go in and have a look at that data before they come to my clinic, and it frees me up to then listen to what they think their priorities are. (Maddy)

## 4 Discussion

This study has provided new insights into the factors nurses consider when recommending mHealth apps to people with chronic conditions. Sometimes, these factors hindered the recommendation of mHealth apps, while at other times, they facilitated app use. Identifying these factors and understanding how they impact behaviours is essential to supporting nurses in maximising the integration of mHealth app technology in usual clinical care.

The findings of this study demonstrated how the accessibility of apps, whether in terms of access to technology (devices, internet, and power) or the appropriateness of mHealth apps to diverse people (older people, non‐English speakers), could be linked to the digital divide. The digital divide is defined as the gap between those with access to devices, internet connection, and software resources and those who do not have access (Van Dijk 2020). Consistent with previous literature, this study raised concerns that limited access to mHealth apps would widen existing socioeconomic disparities and deepen the inequalities in health care (Bucci et al. 2019). The World Health Organization (2022) highlights that ensuring the availability of power and internet access is essential to maximising the benefits of digital interventions. This emphasises the intersectoral nature of health care in that communications infrastructure is essential to delivering equitable digital health interventions. A proposed solution is to create partnerships with public institutions, like libraries and non‐profit organisations, and provide these organisations with specific funding for free internet access to enable people to access health care via technology (Miller et al. 2023; Richardson et al. 2022).

The accessibility of mHealth apps for older people or those with English as a second language was another concern identified in this study that might impede mHealth app recommendation and widen health inequalities. This finding resonates with previous studies, which found that mHealth app developers often overlooked their users' unique lifestyles and sociocultural characteristics, and patients with English as a second language were under‐utilising some beneficial internet tools (Vo et al. 2019). This emphasises the importance of customising mHealth apps to meet the individual needs and sociocultural contexts of patients with chronic conditions (Wang et al. 2024). Involving consumers in the development process is essential to ensure that the apps are effectively tailored to their specific needs, preferences, and diverse cultural backgrounds (Badr et al. 2024).

Findings from this study highlighted that nurses consider the usability of mHealth apps when recommending them to patients. Features like multiple authentications, unnecessary questions, and frequent notifications were seen as frustrating and inappropriate, decreasing consumer engagement. Vaghefi and Tulu (2019) found that while notifications would help patients engage with mHealth apps, having control over these notifications was essential to continued engagement. Strategies like simple, clean, and intuitive interfaces, automatic or minimal data entry, and clear instructions on functionalities likely enhance usability and, subsequently, patient engagement (Deniz‐Garcia et al. 2023; Vaghefi and Tulu 2019). Such features also make it more likely that nurses would recommend these apps to their patients.

Another often overlooked issue within usability is limiting apps to a single chronic condition. Having a mHealth app for every chronic condition would affect the usability of mHealth apps among patients with multiple chronic conditions, eventually decreasing engagement (Deniz‐Garcia et al. 2023). Previous studies have shown that mHealth apps for multiple chronic conditions or polypharmacy tend to have better quality, greater potential for behaviour change, improved medication adherence, and enhanced clinical outcomes (Bricca et al. 2022; Li et al. 2021). Therefore, developing comprehensive mHealth apps that have a multimorbid focus and can help manage multiple chronic conditions at the same time is essential.

## 5 Implications for Education, Policy, Practice and Research

The findings of this study have clear implications. Firstly, health organisations must champion initiatives to ensure equitable access to mHealth technology to bridge the digital divide. This could include providing loan devices for health‐related purposes and considering how they can educate patients on using such technology. Secondly, mHealth apps need to be customised to individual patients' needs, preferences and cultures. This requires ongoing collaboration between nurses, patients and the mHealth app developers. Furthermore, patient feedback is essential to improve the usability and enhance patients' engagement with mHealth apps. lastly, more research is required to understand the patients' perspectives on mHealth apps and how to optimise them to meet their needs and adopt them in both the short and long term.

## 6 Limitations

Since the sample consisted of volunteers, it may be more representative of individuals who are positive or negative about using digital technologies than those who did not participate. However, the participants were from diverse demographic groups, clinical settings, and localities. Another limitation of this study was that interviews were conducted via videoconference due to participants' geographical spread and COVID‐19 restrictions. While this interview mode might limit the development of rapport with participants, the video nature enabled the capture of nonverbal clues. Additionally, video interviews have become more commonplace and acceptable since the outbreak of the COVID‐19 pandemic. While this study set out to explore chronic conditions broadly, further research that focuses on specific disease groups may result in a more nuanced understanding of how mHealth apps could be implemented with specific groups. A final limitation is that while this paper provides the perspective of nurses, it does not capture the perspectives of patients. Further research should ensure that the perspectives of patients are captured to inform the future of app use.

## 7 Conclusion

A deeper understanding of the factors that influence nurses' recommendation of the mHealth apps is crucial to help nurses better integrate them into usual chronic disease management. Key considerations include ensuring patients have reliable access to devices, internet connection, and appropriate software. mHealth apps need to be customised to be relevant to the user's needs, ages, languages, and cultures. Better linking mHealth apps with electronic medical records and health professionals is also essential to optimise health outcomes. Additionally, mHealth apps should have a user‐friendly interface paired with updated, evidence‐based content. Researchers, stakeholders, and mHealth app developers should consider these factors to utilise mHealth apps effectively.

## Author Contributions

Wa'ed. Shiyab led the development of the study, drafted the initial interview schedule, conducted the interview and led the analysis. Kaye Rolls, Caleb Ferguson and Elizabeth Halcomb provided critical feedback about the study design, data collection, analysis and reporting. All authors have contributed to the preparation and review of the paper and agreed on the final version.

## Funding

The authors have nothing to report.

## Conflicts of Interest

The authors declare no conflicts of interest.

## Data Availability

The data that support the findings of this study are available on request from the corresponding author. The data are not publicly available due to privacy or ethical restrictions.
